# The YEASTRACT database: an upgraded information system for the analysis of gene and genomic transcription regulation in *Saccharomyces cerevisiae*

**DOI:** 10.1093/nar/gkt1015

**Published:** 2013-10-28

**Authors:** Miguel Cacho Teixeira, Pedro Tiago Monteiro, Joana Fernandes Guerreiro, Joana Pinho Gonçalves, Nuno Pereira Mira, Sandra Costa dos Santos, Tânia Rodrigues Cabrito, Margarida Palma, Catarina Costa, Alexandre Paulo Francisco, Sara Cordeiro Madeira, Arlindo Limede Oliveira, Ana Teresa Freitas, Isabel Sá-Correia

**Affiliations:** ^1^Instituto Superior Técnico, Universidade de Lisboa, Av. Rovisco Pais, 1049-001 Lisbon, Portugal; ^2^IBB-Institute for Biotechnology and BioEngineering, Centre for Biological and Chemical Engineering, Biological Sciences Research Group, Av. Rovisco Pais, 1049-001 Lisbon, Portugal and ^3^INESC-ID, Knowledge Discovery and Bioinformatics Group, R. Alves Redol, 9, 1000-029 Lisbon, Portugal

## Abstract

The YEASTRACT (http://www.yeastract.com) information system is a tool for the analysis and prediction of transcription regulatory associations in *Saccharomyces cerevisiae*. Last updated in June 2013, this database contains over 200 000 regulatory associations between transcription factors (TFs) and target genes, including 326 DNA binding sites for 113 TFs. All regulatory associations stored in YEASTRACT were revisited and new information was added on the experimental conditions in which those associations take place and on whether the TF is acting on its target genes as activator or repressor. Based on this information, new queries were developed allowing the selection of specific environmental conditions, experimental evidence or positive/negative regulatory effect. This release further offers tools to rank the TFs controlling a gene or genome-wide response by their relative importance, based on (i) the percentage of target genes in the data set; (ii) the enrichment of the TF regulon in the data set when compared with the genome; or (iii) the score computed using the TFRank system, which selects and prioritizes the relevant TFs by walking through the yeast regulatory network. We expect that with the new data and services made available, the system will continue to be instrumental for yeast biologists and systems biology researchers.

## INTRODUCTION

Since its release in 2006, the YEASTRACT (YEAst Search for Transcriptional Regulators And Consensus Tracking; http://www.yeastract.com) database (1) provides to the public up-to-date information on documented regulatory associations between transcription factors (TFs) and target genes, as well as between TFs and DNA binding sites, in *Saccharomyces cerevisiae*. The two groups that compose the YEASTRACT team have contributed to the regular updating of the information therein (the Biological Sciences Research Group, at IST) and introduction of new computational tools to facilitate its analysis (the Knowledge Discovery and Bioinformatics Group, at INESC-ID), considering this task a priority of the joint research (2,3). The YEASTRACT Web site has been accessed by around 8500 researchers, performing nearly 170 000 queries per year during the past 3 years.

Other databases focused on transcriptional regulation in yeast and other model organisms have continuously arisen in the past decade. These databases, including MYBS (4), TRANSFAC (5), RSAT (6), YPA (7) or YeTFaSCo (8), focus most of their analysis and predictive power on the understanding of promoter regions, considering information that includes the occurrence of TF binding sites, but also the accessibility of those putative binding sites, determined among other aspects by nucleosome occupancy data. Besides providing tools for promoter analysis in yeast, YEASTRACT is, to our knowledge, the single information system that offers a complete integration of all the experimentally validated transcriptional regulatory data ever published for *S. cerevisiae*. It has more recently served, on mutual agreement, as the source of most of the transcription regulatory information currently stored in the *Saccharomyces* Genome Database (9).

Beyond offering static data, the major goal of transcriptional regulation databases is to provide reliable tools to predict the transcriptional control of individual genes and of genomic expression reprograming. However, the predictive power of currently available information systems is simultaneously boosted and impaired by the huge amount of available data. In this new release, YEASTRACT is armed with a set of tools to facilitate this analysis and increase the biological relevance of transcriptional control predictions. These include a set of ranking tools that provide different approaches to analyze gene and genomic control of transcription as described later in the text. Among these ranking tools is the recently proposed TFRank method (10), developed to select and prioritize the relevant regulatory players, taking into account an integrated rather than isolated transcriptional control, while walking through the global TF network.

YEASTRACT has been regularly updated, including today 425% more regulatory information than in its previous release. Additionally, a data upgrade was carried out to include specific information on whether the deposited transcriptional associations are positive or negative and on the precise experimental conditions in which the transcriptional association was identified. The new information deposited in the database can be used to filter query results, contributing to obtain predictions with an expected higher biological relevance.

**Figure 1.**
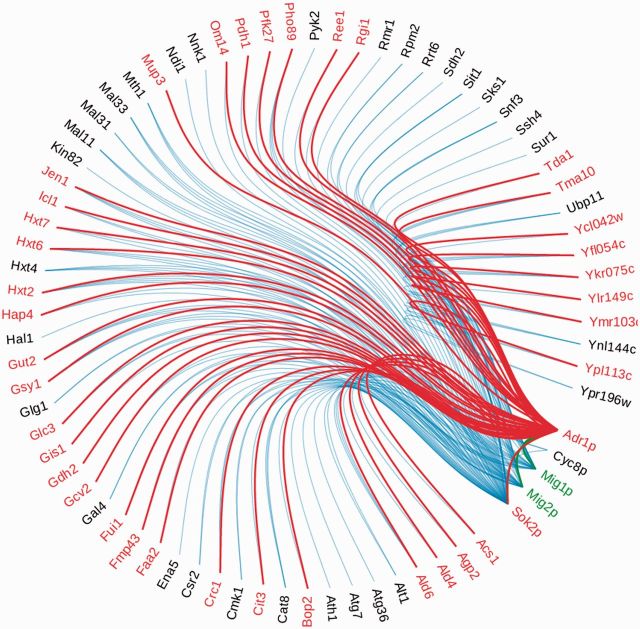
Subnetwork of the top five TFs potentially involved in the yeast response to quinine-induced stress, according to the ranking based on the enrichment of TF targets in the user’s gene list, is displayed, illustrating the new visualization tools available at YEASTRACT. The TFs are isolated from the target genes in the right-down corner of the figure. In red are the targets of Adr1, which appear on selection by the user, whereas in green are the TFs that are regulators of Adr1.

Finally, new visualization tools have been made available to enable an interactive global view of genomic scale regulatory networks (illustrated in Figure 1).

## DATA UPGRADE

Throughout the period of 7 years that has gone by since the first YEASTRACT release (1), the information in the database has been regularly updated (2,3). Since the previous release, in 2011, >158.000 regulatory associations were added to the database, which presently contains 206.299 regulatory associations between genes and TFs.

Furthermore, an upgrade in the quality and classification of the stored data was achieved. The 1.337 articles that underlie the data gathered so far were manually revisited by the YEASTRACT curators. Information on the experimental basis of the associations between TFs and target genes was searched for and added to the database. The underlying experimental evidence was also collected and classified as either ‘DNA Binding’ or ‘Expression Evidence’ (11). ‘DNA Binding Evidence’ was considered to be provided through experiments directly measuring the binding of the TF to the promoter region of the target gene (e.g. Chromatin ImmunoPrecipitation (ChIP), ChIP-on-chip, ChIP-seq and Electrophoretic Mobility Shift Assay) or the analysis of the effect on target-gene expression of the site-directed mutation of the TF binding site in its promoter region, as strongly suggesting an interaction of the TF with that specific target promoter. ‘Expression Evidence’ classification was attributed to experiments such as the comparative analysis of gene expression changes occurring in response to the deletion, mutation or overexpression of a given TF, based on experimental techniques that include northern blotting, quantitative RT-PCR, microarray analysis or expression proteomics. Based on this classification, YEASTRACT contains 41.693 regulatory associations based on DNA binding evidence and 172.814 on expression evidence, with some overlap. Classification according to environmental condition was also obtained and >730 different environmental conditions in which data were obtained were grouped into 10 clusters, including stress, oxygen availability, unstressed log-phase growth (control), nitrogen source quality/availability, carbon source quality/availability, ion/metal/phosphate/sulfur/vitamin availability, cell cycle/morphology, lipid supplementation, complex industrial media and *in vitro*. Each of these clusters is composed by sub-clusters to enable a finer filtering of the existing regulatory associations.

This classification, based on different types of experimental evidence or environmental conditions, permitted the inclusion of additional constraints in the queries GenerateRegulationMatrix, SearchForAssociations, RankByTF, SearchForTFs and SearchForGenes. For example, it is now possible to group a list of genes, e.g. the genes found to be upregulated in a given microarray analysis, based on the TFs that activate the expression of the genes in the data set, and excluding those TFs that exert a repressive effect on those same genes, or based on the regulatory associations known to occur under a specific environmental condition, closer to the one used to obtain the data set under analysis. This can prove to be extremely useful to narrow down a given search or grouping to the TFs that are potentially more relevant.

## TF RANKING TOOLS

Since its first release, the YEASTRACT database has incorporated analysis tools for investigation of the transcriptional regulation of genes involved in a particular biological response. Researchers are often interested in looking for shared regulation of the target genes, which can unravel potential interactions between regulatory players and ultimately provide clues on the regulatory pathways that underlie the observed transcriptional response. A popular approach to unveil co-regulation consists in identifying which TFs regulate the genes of interest and then listing the genes regulated by the same TF, based on known regulatory interactions. This search has been available since the first release of YEASTRACT (1). Alternatively, plausible co-regulation can be derived through the identification of common regulatory motifs on the promoter regions of the target genes, using pattern matching or motif finding algorithms. These techniques have been associated with the YEASTRACT database since its second version (3).

In this new version, we introduce two new ranking tools that enable the prediction of the relevance of TFs in a more meaningful way. One is relatively simple and provides ranking based on the enrichment of TF targets in the user’s gene list, considering the ratio of genes they target in the data set when compared with the equivalent ratio in the genome. A similar approach is used by many genome-wide data analysis tools to compute the enrichment of Gene Ontology terms for a given set of genes [e.g. GoToolBox (12) or FunCat (13)]. When ranking TFs, the use of the enrichment-based ranking reduces the preponderance of TFs with large and complex regulons and helps to highlight TFs that do not yield many regulations in the genome, but may play a key role in a specific response. This is particularly relevant considering the fact that the number of known targets for each TF in yeast varies dramatically, from 1 for Asf1 or Mdl2 to 4776 for Ace2. For example, when applying the ‘Rank by TF’ tool to the genes upregulated on exposure to quinine (14), the enrichment-based ranking of TFs reported several TFs (Mig1, Mig2 and Adr1) involved in glucose derepression as being among the top ranking TFs and potential key regulators of the yeast response to low-inhibitory concentrations of quinine (Figure 1), whereas these TFs would only appear after other more general regulators known to play a role in the regulation of yeast response to several environmental stresses if the genes were ranked according to the number of genes they regulate in the data set. Significantly, yeast adaptation to quinine was shown to involve a glucose limitation response, probably as a consequence of glucose uptake inhibition by the drug (14).

**Figure 2.**
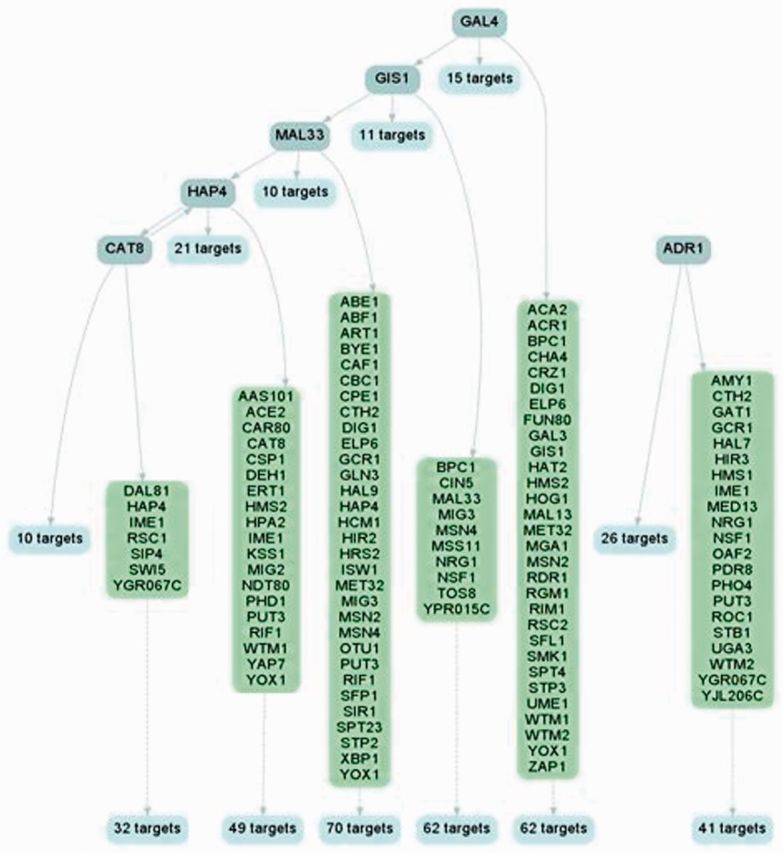
Subnetwork of the top six TFs potentially involved in the yeast response to quinine-induced stress, according to the TFRank algorithm. Light blue boxes show the number of quinine-induced genes reportedly regulated by the top TFs (dark blue) or by a layer of other TFs (green) controlled by the top TFs.

An additional ranking method that was included in the database is TFRank (10). This algorithm goes beyond classical approaches by taking into account the relevant parts of the regulation network when ranking the regulators of a transcriptional response. This method exploits every regulatory path containing genes of interest to achieve prioritization of regulators by computing a relevance measure that reflects their contribution within the regulatory network under study. Advantages of the TFRank algorithm further include ability to consider multiple levels of regulation and interactions between TFs in an integrated rather than isolated-per-TF network analysis perspective. The relevance measure or ranking score can be computed using different parameters that enable a fine tuning of the analysis being made. In YEASTRACT, the TFRank algorithm provides the user the option to customize two additional properties: the heat diffusion coefficient and regulation weights. The first property controls the range of influence of the regulatory cascade in the network allowing the user to regulate its preference for more global or local regulatory players. Regulation weights are attributed according to the new regulatory associations filters set by the user. For instance, one can give priority to regulations with expression evidence where the TF acts as an activator over all other regulations. As an example of the application of the algorithm, we analyzed the same set of genes upregulated on exposure to low-inhibitory concentrations of quinine (15), which is a relatively simple data set and well-characterized biological response and thus useful to illustrate the value of the algorithm. In this analysis the TFRank algorithm was used with the diffusion coefficient set to a lower value (*t = *0.25) to favor proximal regulators, presumably more specific to the biological response under study. In this case, TFRank indicated Adr1, Hap4, Gal4, Mal33, Gis1 and Cat8 (Figure 2) as the most relevant mediators of the yeast transcriptional response to quinine. Notably, all these TFs were found to be upregulated in response to quinine stress and are known to play a role in yeast adaptation to alternative carbon sources. This is in agreement with the fact that quinine induces intracellular glucose limitation. In clear contrast, the top TFs obtained based on the percentage of documented regulatory associations targeting the upregulated genes in the quinine data set are associated with more general cellular responses, and none of them was found upregulated in response to quinine stress (15). Even when compared with the TF enrichment tool described earlier in the text, TFRank highlights a higher number of glucose limitation TFs and proposes the network frame shift in which they operate. It is possible that the analysis of more complex biological responses might require specific manipulation of additional parameters of the algorithm to optimize the information that can be obtained from the data. This can be achieved by using the original version of TFRank (10).

## FURTHER AVAILABLE RESOURCES

In prior versions of YEASTRACT, the available queries only offered the selection of potential—based on the occurrence of TF binding sites in the target gene promoter regions—or documented transcriptional associations. Based on the data upgrade, it is now possible to filter most queries taking into account further criteria.

**Figure 3.**
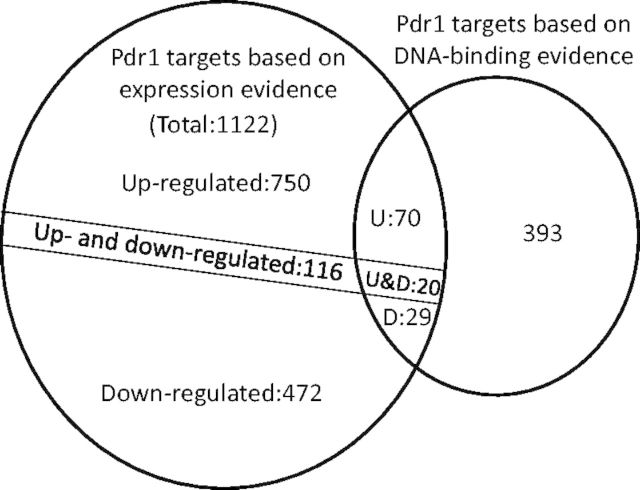
Network of Pdr1 target genes, highlighting the importance of selecting appropriate filters—namely those regarding the underlying experimental evidence and mode of action of the TF—to obtain good predictions on regulatory control.

For instance, the user may now select transcriptional associations for which there is DNA binding evidence, those for which there is expression evidence or for those for which there is simultaneously DNA binding and expression evidence. This selection may increase the degree of confidence on the actual direct transcriptional control under analysis. This querying hypothesis may be meaningful because for many regulatory associations there is only a small overlap between TF target genes established by each experimental approach. For example, the multidrug resistance TF Pdr1 has been shown to bind to 393 target promoter regions, while affecting the expression of 1122 target genes. However, the intersection of the genes whose promoter regions are known to be bound in some conditions by Pdr1 with those whose expression changes in the dependency of Pdr1 expression or activity shows that only 116 genes appear to be directly and effectively controlled by Pdr1 (Figure 3).

An additional filtering option associated to several queries in YEASTRACT is based on the selection of the environmental condition in which the TF-target gene association was uncovered. This may be an important filter because it is clear that each TF-target gene association is highly dependent on the environmental condition that will trigger specific signaling pathways instead of the whole network. Thus, the observation that a given set of genes is predicted to be controlled by a TF that is not active in the conditions of the experiment under analysis may be misleading.

Finally, all queries can also be filtered based on the mode of action of the TF in their target genes, either as activator or repressor. Taking the Pdr1 case again as an example, it is possible to observe that within the regulatory associations deposited in YEASTRACT, 750 targets are found to be upregulated by Pdr1, whereas 472 are downregulated by this TF, 116 of which can be either up- or downregulated by Pdr1 depending on the environmental and experimental setup (Figure 3). Although Pdr1 is only recognized as an activator, the fact is that its expression apparently has an indirect repressive effect on numerous genes. Significantly, upregulated genes include mostly those with a traditionally attributed role in multidrug resistance, including multidrug transporters or genes involved in the control of membrane lipid composition, whereas those downregulated are associated with more general processes such as regulation of transcription and translation and ion homeostasis, suggesting that the repressive effect exerted by Pdr1 is indirect. Consistent with the notion that Pdr1 is a transcriptional activator, it is interesting to point out that most of the Pdr1-target gene associations for which there is both DNA binding and expression evidence correspond to upregulated genes. Furthermore, if this search is further restricted to Pdr1 targets identified under stress conditions, it is possible to retrieve >75% of those associations for which there is simultaneous DNA binding and expression evidence. This example highlights how the use of the new filters provided by YEASTRACT can improve the quality of the prediction of which specific transcription regulatory networks may actually be working in specific conditions.

All previous and updated regulatory data on YEASTRACT continue to be provided by a set of web service resources accessible through a RESTful Application Programming Interface (API). This API empowers users with the possibility to access YEASTRACT according to their specific needs, by developing client-side code to query, explore and retrieve curated data. Even though the regulations web service resource is capable of retrieving all the updated data, it does not supply information regarding the associated environmental condition and association type, as the API result fields did not suffer any change.

## FUTURE DIRECTIONS

The YEASTRACT team is committed to continue to offer updated, reliable and complete information on the field of transcriptional regulation in yeast to the international community of yeast and systems biologists. Furthermore, continuous improvements of the provided tools will be made available, in response to the requests and needs of its users. Particular focus will be given in the future to the extension of the database to the *S. cerevisiae* pan-genome and to other yeasts of biomedical and biotechnological interest, in a comparative genomics approach.

## FUNDING

FCT-Fundação para a Ciência e a Tecnologia, under the contracts [Pest-OE/EQB/LA0023/2011_research line: Systems and Synthetic Biology, ERA-IB/0002/2010, PTDC/EIA-EIA/111239/2009, PTDC/EIA-CCO/118522/2010] and postdoctoral and PhD grants (to P.T.M., J.F.G., J.P.G., S.C.S., T.R.C., M.P., C.C. and S.C.M.). Funding for open access charges: FCT-Fundação para a Ciência e a Tecnologia.

*Conflict of interest statement*. None declared.
